# Prevalence of Asbestos-Related Disease Among Workers in Sri Lanka

**DOI:** 10.5334/aogh.2575

**Published:** 2019-07-15

**Authors:** B. Aseni Wickramatillake, Menaka A. Fernando, Arthur L. Frank

**Affiliations:** 1 University of Mortuwa, LK; 2 University of Technology, LK; 3 No Affiliation, LK; 4 Dornsife School of Public Health of Drexel University, US

## Abstract

**Background:**

Asbestos products are manufactured and used in Sri Lanka in the construction and automobile industries.

**Objective:**

To determine radiologically if exposure to asbestos caused lung disease among workers handling asbestos products, and to generate data in Sri Lanka where no such data exist due to poor reporting and a poor surveillance system.

**Methods:**

Following ethics approval and written consent plain chest X-rays and exposure data were obtained in 230 workers in asbestos manufacturing, building construction, building demolition, tsunami debris cleanup, and other trades. The assumption was that all exposed workers were exposed to chrysotile. Participants were from provinces with asbestos factories, and where tsunami cleanup had occurred.

**Findings and Conclusions:**

Radiological findings of the 230 participants showed lung fibrosis in 7% (16 cases), and other non-asbestos-related lung conditions. Of the 16 fibrosis cases, none were in manufacturing workers, one in a construction worker, six in tsunami workers, six in demolition workers, and three cases in others. Globally, Sri Lanka has one of the highest consumptions of chrysotile asbestos per capita. This first known study documenting asbestos disease in Sri Lanka is clearly a limited, self-selected group of workers studied with obvious limitations. The prevalence of asbestos-related lung disease among tsunami and demolition worker indicates that a risk exists for asbestos material already in use in Sri Lanka. Hence a significant concern is the safety of asbestos demolition workers and cleanup workers exposed to asbestos debris from major natural disasters such as hurricanes, tornados, typhoons, and tsunamis.

## Introduction

Asbestos minerals are found in two groups of six fibres called serpentine and amphibole. Chrysotile alone belongs to the serpentine group [1] and is widely used in Sri Lanka. The International Agency for Research on Cancer (IARC) has classified asbestos, including chrysotile, as carcinogenic and none of these asbestos fibres has an identified threshold risk for cancer [2].

Inhalation of asbestos fiber can lead to a variety of respiratory pathologies including asbestosis, lung cancer and mesothelioma.

In the past, these asbestos minerals have been mined throughout the world by many countries. The global mine production of asbestos has been on the decline since 2007 from 2,200,000 metric tons to 2,000,000 metric tons in 2012 [3]. Data extracted from International Ban Asbestos Secretariat (IBAS) in 2018 shows Russia, China, Brazil and Kazakhstan were the 4 main countries producing asbestos fibres for global consumption in 2016. Russia produces the majority of the total production. Since 2016, Brazil has closed down all of its asbestos mines. By comparison to year 2012, in 2016 the global production of asbestos has further decreased mainly due to the decreased production by Russia. A major producer of asbestos in the world, Canada, closed most of its production mines in Quebec in 2012 and the Government of Canada banned asbestos in 2018 after the unscientific and unethical governmental asbestos policy was repeatedly challenged by a group of scientists in Quebec [4]. Colombia voted in 2019 to stop the mining, export, and use of asbestos as well.

**Figure 1:**
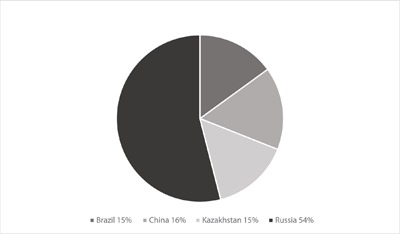
Asbestos: World Production By Country 2016.

Due to its various properties, asbestos has been a popular material in construction, shipbuilding and repair and automotive products [5]. Due to the accumulating evidence of asbestos related health problems many countries have already banned the use of asbestos. However, some low- and middle-income countries in Asia, South America and the Middle East continue to use asbestos, as has the United States. In Sri Lanka, between 2012 and 2016, over 200,000 metric tons of asbestos was consumed. As estimated by IBAS, comparing 2016 to 2012, a large number of countries have reduced their asbestos consumption except for 3 countries. It is regrettable that Sri Lanka is among the three countries that have increased its asbestos use. The other two countries are Bangladesh and the Philippines. Bangladesh has been estimated to have a five-fold increase in year 2016, when compared to 2012. The populations of Bangladesh and Sri Lanka in year 2016 are 163 million and 21.2 million respectively but use in Bangladesh for those same years was only about 44,000 metric tons. It is notable that Sri Lanka is consuming four times greater quantities of asbestos per capita among a population seven times less than in Bangladesh. In year 2016, the largest asbestos consumer in the world, India, used an estimated 233 metric tons per million of its population (1.324 billion), while in Sri Lanka that figure is 235 metric tons per million individuals. It is clear that Sri Lanka consumes large amounts of asbestos per capita.

Despite interest and initiatives to enforce a global ban by organisations such as World Health Organization (WHO), the International Labour Organization (ILO) and the Collegium Ramazzini, pro asbestos lobbies globally continue to claim that there is little or no risk of exposure to chrysotile, which is scientifically unjustified [6,7].

Identified occupational exposures to asbestos in Sri Lanka are from asbestos roofing sheet manufacturing plants; building construction and demolition; 2004 tsunami clean-up work; changing of vehicle brake pads; working in boiler rooms; transporting and handling of asbestos products; dockyards where ship repairs are done; and dwellers in close proximity to asbestos manufacturing plants. Environmental exposure occurs when brittle asbestos is handled and when people dwell in housing with brittle asbestos roofing products.

Due to its commercially viable properties, chrysotile asbestos roofing sheets are widely used in Sri Lanka. Since the 1960s, Sri Lanka has been increasingly using corrugated asbestos cement sheets and other asbestos-containing materials in the construction industry. Chrysotile asbestos is the most widely used type in Sri Lanka in asbestos cement building material. Chrysotile is mixed with cement to produce the roofing sheets [8]. A change in the Sri Lankan Government in 2015 brought about a ban on asbestos use by the year 2018, later changing it to 2024. However, due to international political pressure from producers of asbestos and adverse effects on international trade the ban on asbestos may not actually occur.

According to the Ministry of Industry and Commerce, Sri Lanka has been among the leading importers of asbestos, absorbing 6% of the global asbestos imports in 2015 [9]. Sri Lanka imports the majority of its chrysotile asbestos from Russia. Russia and Iran have been the main importers of Sri Lankan (Ceylon) tea, which draws in large amounts of foreign revenue to the country and puts pressure on trade relationships.

The epidemiological status of asbestos related health issues in Sri Lanka is not well known despite mandatory CXR investigations required of employees in the asbestos roofing sheet manufacturing plants. According to employees and workers unions, results of such tests are not disclosed to the employees with medical files locked away under the strict control of the management. Underreporting occurs due to ignorance of dangers, incomplete investigations procedures, lack of registering and recording of diagnosed cases. A false sense of security is developed in the minds of the Sri Lankan communities due to the lack of evidence or data regarding asbestos related illnesses. There appears to be no data in the open scientific literature about asbestos diseases among Sri Lankans.

## Methodology

The aim of this descriptive study was to determine, by radiological investigation, whether exposure to asbestos dust at workplaces caused lung disease among workers handling asbestos products. Ethics approval was obtained from the Faculty of Medicine, University of Colombo, Sri Lanka. Written consent was obtained from each participant. Counseling was provided to participants who were distraught due to their asbestos exposure concerns.

Due to a lack of occupational health surveillance system in Sri Lanka, statistical data is almost non-existent. Underreporting, incorrect pathological diagnoses, and inadequate emphasis by the medical professionals on the occupational history may be contributory factors.

This study was conducted by screening 230 self-selected asbestos exposed workers to obtain the asbestos-related disease status through radiological changes on plain CXR. Five of the nine provinces in Sri Lanka were involved, and two questionnaires were completed by participants prior to taking a chest X-ray. The provinces involved included those with Sri Lankan asbestos factories and where tsunami cleanup had taken place. The radiological investigations were done at medical centers convenient to participants in each province. Chest radiographs were independently read according to the 1980 International Classification of Radiographs of Pneumoconiosis of the International Labour Organization (ILO) by two qualified and experienced readers. The study population consisted of (1) workers employed in asbestos manufacturing plants; (2) building construction workers; (3) persons involved in large scale tsunami debris clearing work; (4) persons engaged in demolishing old buildings; and (5) past and present workers of asbestos related occupations (other than in asbestos manufacturing plants) such as: workers involved in decorative molding with asbestos; vehicle mechanics working with brake pads; and dockyard workers.

Participants were from five of the nine provinces (Western, North Western, Central, Eastern, and Southern) in Sri Lanka where asbestos factories are located, tsunami clean-up had taken place, and construction industry was booming. North and North Central provinces were not included in the study due to logistics and security reasons. UVA and Sabaragamuwa provinces were omitted due to minimal amount of construction and demolition work in these areas. The radiological investigations were done at medical centers convenient to participants in each province due to the inconvenience of travelling to one central location. Basic plain chest radiographs were taken in seven government-approved centers located in the North Western (Chilaw and Negombo towns), Western (Moratuwa town and Colombo city), Southern (Galle town), Central (Kandy city), and Eastern (Batticaloa town) provinces. A questionnaire was completed by the participants on age, sex, smoking history, work history, and exposure history.

Chest radiographs were independently read according to the 1980 International Classification of Radiographs of Pneumoconiosis of the International Labour Organization (ILO) by two qualified radiologists. In Sri Lanka, chest radiographs are commonly done as a primary investigation due to the low cost, hence the radiologists are well experienced.

The mean age range of the participants were from 17 years to 70 years with a mean age of 40.5 years. 10% of the participants were females. Of the 230 participants, 25 (10.9%) were asbestos manufacturer workers; 146 (63.5%) were building construction workers; 20 (8.7%) were building demolishers (inclusive of tsunami debris clearing workers); and 36 (15.7%) were past and present workers of asbestos-related occupations.

Workers involved in clearing tsunami debris were considered as a cohort group with possible follow up in future as they may not show changes in the radiological images due to the short latency since exposure. No workplace measurements were taken, nor assessment of fiber type exposure, though most, if not all, of asbestos used would have been chrysotile.

## Results

A total of 230 workers participated in the study of which 207 were males (mean age 39.7 years, age range between 17-70 years) and 23 females (mean age 46.5 years, age range between 23–65 years). 47% of males were active or ex-smokers while no females smoked.

Among the 230 participants 7% (16 cases) displayed lung fibrosis consistent with prior asbestos exposure. By exposure duration, the number of cases presenting lung fibrosis was as follows: 13 out of 16 people were exposed to asbestos at work for more than 10 years. Among the 16 diagnosed with fibrosis, 4 were smokers and only 1 was a female.

Lung fibrosis was detected in one of 149 construction worker, six of the eight tsunami debris clearing workers, six of twelve demolition workers, and three workers in other industries. 27 participants showed lung conditions other than lung fibrosis on the chest x-rays. Radiologically, 15 participants had cardiomegaly, 5 had lung nodules, 2 had bronchiectasis, and 5 had other conditions such as emphysema.

**Table 1:** Documents fibrosis cases by duration of exposure.

Number of Cases	Exposure Duration

1 case	0–5 years
2 cases	6–10 years
4 cases	11–15 years
2 cases	16–20 years
5 cases	21–25 years
1 case	26–30 years
1 case	>30 years

## Discussion

Sri Lanka manufactures its own asbestos cement roofing sheets in manufacturing plants. As in many other developing countries, Sri Lanka’s continued use of asbestos in the construction industry is of concern. In Sri Lanka the construction industry employs a significant number of people due to infrastructure development and the increase in building of multi-story apartments. There are no adequate regulations on the use and disposal of asbestos material. Manipulation, such as cutting asbestos roofing sheets to size and drilling holes in the sheets to insert screws, are all done at the construction site; not only exposing workers engaged in such activities, but also exposing other employees. There are no engineering controls such as wet cutting or local exhaust ventilation systems used during such activities. Work practices and administration of asbestos use is not monitored due to the lack of awareness among administrators and employees. No safety goggles, respirators, or protective clothing are worn by these workers, except for some covering their nose and mouth with a piece of cotton fabric.

This is the same practice used during demolition or discarding of asbestos roofing sheets. Not only are the demolition and repair workers exposed to asbestos dust particles, but the waste products are mixed with general waste exposing many others to asbestos dust.

During the Tsunami of December 2004, many people assisted or worked in the clearing up of debris, which exposed them to large quantities of broken asbestos products. Due to the long latency of asbestos-related diseases some of the participants may not yet show any changes on CXRs. Disease may occur later in their lives, but some were seen now.

A majority of both employers and employees do not take adequate precautions when working with asbestos related products. This may be due to lack of awareness of the dangers and the uncertainties of the risks from asbestos.

The search for available records from the National and other General Hospitals in Sri Lanka on asbestos-related illness also did not produce any valuable information. All communication with radiologists, physicians, respiratory physicians, and pathologists stated that although some statistics are available on lung disease and lung cancer, it cannot be in anyway attributed to asbestos. Individual patient data were not obtained for this study. A consultant oncologist of the National Cancer Institute stated that the small number of mesotheliomas found in Sri Lanka could be because some mesotheliomas may have been misdiagnosed as adenocarcinomas. This may be contributed by the lack of advanced diagnostic methods, underreporting, and lung surgeries being carried out without complete pathological diagnoses.

The National Cancer Registry in Maharagama and regional cancer units in Kandy and Galle have limited information on lung cancer; none specific to asbestos. Little research on asbestos exposure has been done in Sri Lanka. (1) There is a dissertation in the National Institute of Occupational Safety and Health – Sri Lanka (NIOSH-SL) library [10]; (2) a study done in three different scenarios representing different stages of chrysotile-cement products and the suspected risk of exposure [11]; (3) a comparative analysis of chrysotile fibre cement roofing sheets and the proposed alternative fibre roofing sheets [12]. None of these papers were published in peer reviewed journals. Some of these studies were done at the request of the Chrysotile Information Centre in Sri Lanka. Some studies have to be reviewed with caution as some companies are still trying to create doubt, as is done elsewhere in the world, regarding the harm of asbestos [13]. The Indian government agency carrying out an asbestos study had been designed and assisted by the asbestos industry, with potential for inaccuracies [3]. Similarly, the Sri Lankan government should avoid conducting any studies with links to lobbyists from the asbestos industry. All such studies, such as ours, are best done by independent scientists.

A lack of data about asbestos disease in Sri Lanka may engender a false sense of assurance about lack of disease. There may be many reasons for not reporting, such as fear of job loss [14].

As research has documented in South Africa, England, and Finland, neighbourhood exposure [3] can be a significant problem in Sri Lanka due to poorly controlled handling of asbestos products in manufacturing plants as well as other construction and demolition sites in urban areas. In interviews of neighbours in an area of asbestos roofing sheet manufacturing plant, the neighbours complain of fine dust accumulating in their houses despite daily cleaning. People known as “bystanders” who are engaged in other work in close proximity to areas with asbestos work or families of workers working with asbestos products are likely to be exposed to asbestos fibres through air-borne particles or contaminated clothing [3].

Deaths in Sri Lanka are generally not documented in relation to employment. In Sri Lanka with the significant infrastructure development and increase use of asbestos, the country may experience a significant disease burden in the near future. There are also implications for workers and others who clean up asbestos-containing materials after episodes of hurricanes, tornados, and typhoons worldwide.
